# Prognosis in Tubercular Peritonitis

**Published:** 1903-09-26

**Authors:** 


### Prognosis in Tubercular Peritonitis.

It is always an important advance in clinical knowledge when reasons can be submitted to show that the prognosis in any particular pathological condition is less gloomy than current doctrine describes it. So long as the anticipation is held that all therapeutic measures will prove of no avail these measures inevitably lack vigour. But with a prospect of success, treatment in a corresponding degree gains in intelligence. A ready illustration of these statements is provided by the present hopeful attitude of the profession towards phthisis pulmonalis, as contrasted with the note of despair of a previous generation. The recognition of the fact that tubercular disease of the lung does, in numerous instances, undergo arrest, has both encouraged the patient and directed the physician. Some recent discussions suggest that an unduly pessimistic attitude is largely held and taught in reference to tubercular peritonitis. Short of surgery, we are told, there is in this disease little or no chance of cure. Indeed, the announcement is made that until the surgeon came upon the scene the possibility of cure was not even entertained. Now no one conversant with the facts will be anxious to deny that active surgical interference has in certain types of tubercular peritonitis proved decidedly successful. But there is a wide interval between this proposition and the suggestion that, in every case, unless surgery can save the patient it is time to abandon hope. Neither is it correct to affirm that prior to the advent of the surgeon the prognosis in tubercular peritonitis was invariably painted in the darkest possible colours. It may, however, be allowed that the practice of abdominal incision has both added a further shaft to the therapeutic quiver and has shown that relatively slight disturbances of intra-abdominal conditions may lead to an arrest of the tubercular process. In this latter fact may be found much ground for encouragement. For if the mere incision of the abdominal wall and evacuation of fluid from the peritoneal cavity may abolish the activity of the tubercle bacilli, it is natural to expect that other modifications of their environment, such as may be produced by medicinal treatment, may be followed by the same result. There are other circumstances which may be urged in support of the suggestion that tubercular peritonitis ought not to be clouded with a prognosis of hopelessness. First, to state it for what it is worth, there is the argument from analogy. If tubercular disease in the thorax may in favourable circumstances come to a happy termination, it is reasonable to anticipate that the same will be true of the disease when it develops within the abdomen. There is nothing in the nature of things to render improbable in the one case what experience has shown to be fully possible in the other. Again, there are certain post-mortem facts to be considered. Thus in a puckered and calcified cicatrix at the apex of a lung it tells of a sustained victory over the bacillus of tubercle. A similar inference must be attached to the discovery of caseous and calcareous mesenteric glands. These are not very uncommon in the bodies of adults who have died from various diseases and without any suspicion of tuberculosis. Thus in the analogy of pulmonary phthisis, in certain surgical successes, and in the facts of post mortem examination, may be found reasons for relieving, at least in some degree, the gravity of the prognosis in cases of tubercular peritonitis. Last of all, and towards the same end, may be urged the results of clinical experience. Observers whose knowledge and accuracy are beyond reproach have from time to time claimed success in the treatment of a certain proportion of cases of this disease. There is no question here of a mere nominal diagnosis of tabes mesenterica on the basis of simple abdominal distension and more or less diarrhœa. The reference is to physicians of admitted competence with large opportunities for observation. Of course every case which escapes the post-mortem table is open to the suspicion of a mistaken diagnosis. But evidence may be sufficient for the suffrages of reasonable men, though it fall short of actual demonstration. And it is beyond dispute that in instances not a few, symptoms and signs indicative of tubercular peritonitis have been known to subside and the patient to regain a full measure of health. With the detailed application of measures of treatment we are not now dealing. Our purpose is to controvert a doctrine of despair and to establish the proposition that in many cases of tubercular peritonitis there is a fair and reasonable prospect of successful practice.

